# Green Fluorescent Diamidines as Diagnostic Probes for Trypanosomes

**DOI:** 10.1128/AAC.02024-13

**Published:** 2014-03

**Authors:** Federica Giordani, Manoj Munde, W. David Wilson, Mohamed A. Ismail, Arvind Kumar, David W. Boykin, Michael P. Barrett

**Affiliations:** a Department of Chemistry, Georgia State University, Atlanta, Georgia, USA; b Department of Chemistry, College of Science, King Faisal University, Hofuf, Saudi Arabia; c Wellcome Trust Centre for Molecular Parasitology, Institute of Infection, Immunity and Inflammation, College of Medical, Veterinary and Life Sciences, University of Glasgow, Glasgow, United Kingdom

## Abstract

Light-emitting diode (LED) fluorescence microscopy offers potential benefits in the diagnosis of human African trypanosomiasis and in other aspects of diseases management, such as detection of drug-resistant strains. To advance such approaches, reliable and specific fluorescent markers to stain parasites in human fluids are needed. Here we describe a series of novel green fluorescent diamidines and their suitability as probes with which to stain trypanosomes.

## TEXT

Early diagnosis of human African trypanosomiasis (HAT) (1) is paramount for favorable prognosis, but it is difficult to achieve in the field (2). Conventional light microscopy, in particular, is insensitive and time-consuming. Deployment of better diagnostics, together with more effective drugs, will be essential to eliminate the disease as a public health problem.

The development of portable and efficient light-emitting diode (LED)-illuminated microscopes (3–5) allows fluorescence microscopy to offer improvements in detection. Its use in the diagnosis of other tropical diseases has already been shown to increase the sensitivity and rapidity of sample preparation and screening (6, 7). A similar approach would also be useful in diagnosis of animal trypanosomiasis, which suffers limitations similar to those of diagnosis of HAT. The diagnostic fluorescence tests developed for HAT (8, 9) use acridine orange to stain trypanosomes. This is a cheap but aspecific fluorophore, which can label the nucleic acids of any cell in the specimen, and as a DNA intercalator, it poses potential risks to laboratory technicians (although, based on available data, the IARC inserts it in group 3, as not classifiable as to its carcinogenicity for humans).

The aim of this work was to synthesize and evaluate a series of fluorescent diamidines as targeted diagnostic probes for trypanosomes. Diamidines (10, 11) appeared promising since they are rapidly internalized by Trypanosoma brucei via specific surface transporters, including the aminopurine P2/*Tb*AT1 transporter (12–14), and several of them are fluorescent (11, 15, 16). The UV emission of DB99 was previously exploited in our laboratory to develop a simple assay for detection of drug-resistant strains lacking P2 (17). Unfortunately, although LED fluorescence microscopes are being increasingly used, UV LED-illuminated instruments are not yet widely available, most fluorescence biological work being carried out at longer wavelengths.

(Biological aspects of this work were conducted by F. Giordani in partial fulfillment of the requirements for a Ph.D. from the University of Glasgow, Glasgow, Scotland, 2011.)

By addition of a series of ultraconjugated ring structures in the linker between the two amidine groups (containing the recognition motif for the parasite's P2 transporter), we generated a series of new diamidines with longer emission wavelengths than the parental UV-emitting DB75 (chemistry data are presented in the supplemental material). Spectral measurements confirmed that all novel dications emitted in the green wavelength range (Table 1). As expected for diamidines (11), the new molecules had a high affinity for DNA. Values of DNA binding by thermal melting (Δ*T_m_*, measured as described in reference 15) revealed the binding affinity to be greater than that of pentamidine (12.6°C) but lower than that of DB75 (25°C) (Table 1).

**TABLE 1:**
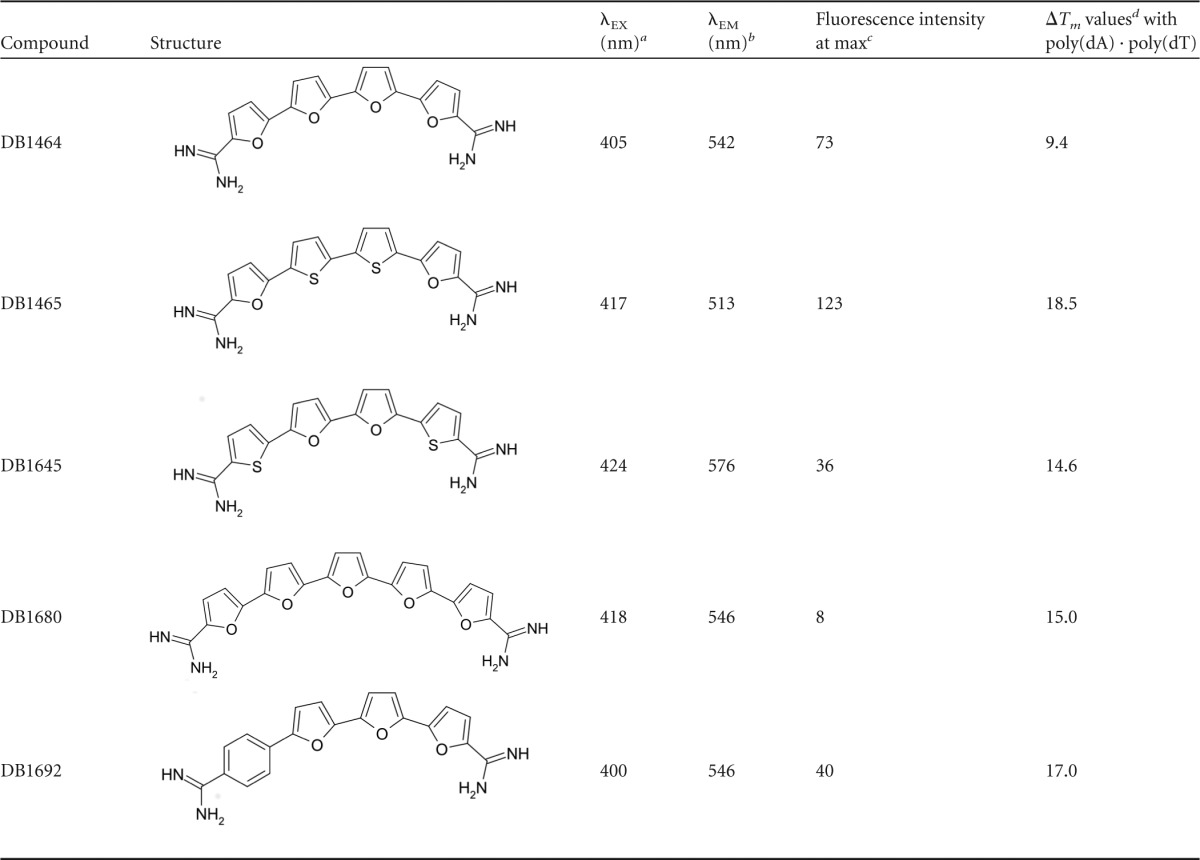
Structures and physical properties of the fluorescent diamidines

a Excitation wavelength.

b Emission wavelength.

c max, maximum.

d Melting temperature of the compound-DNA complexes as compared to values for free DNA. *T_m_* values are in degrees Celsius and have an error of ±0.5°C.

The dependence of the compounds on P2 transport for uptake into trypanosomes was confirmed by an alamarBlue assay (18) performed on three Trypanosoma brucei brucei strains: a wild-type line (bloodstream Lister 427), a line not expressing P2 (*tbat1*^−/−^) (19), and a derived clone, in which high-affinity pentamidine transporter (HAPT1, now identified as an aquaglyceroporin [20]) activity is also lost (B48) (21). Compound DB1692 showed the greatest dependency on P2 for transport (50% inhibitory concentration [IC_50_] 25-fold higher for the *tbat1*^−/−^ line than for the wild type), followed by DB1645 (16-fold higher), DB1465 (10-fold), and DB1464 (8-fold) (Table 2). Data using the B48 line showed that HAPT1 activity also contributed to the accumulation of DB1645 and DB1465. In contrast, the addition of a single furan ring in the linker of DB1680 highly affected the rate of uptake of this compound through the P2 transporter, and further loss of HAPT1 did not affect its toxicity, suggesting other routes of internalization (e.g., other carriers or endocytosis). Equally noteworthy is the potent *in vitro* activity shown by these new compounds against the wild-type line, especially by DB1464, which had an IC_50_ close to that of pentamidine. This indicates that these molecules may be attractive candidates to pursue as possible leads against HAT, provided that ADME (absorption, distribution, metabolism, and excretion) characterization demonstrates good pharmacokinetic properties and rules out potential toxicity issues previously observed for some molecules belonging to this class.

**TABLE 2:** *In vitro* trypanocidal activities of the five diamidines against T. b. brucei S427 wild-type and derived cell lines^*a*^

Compound	IC_50_ [nM] (± SEM) for:		RF^*b*^	IC_50_ [nM] (± SEM) for B48	RF
	Wild type	*tbat1*^−/−^ line			
DB1464	0.9 ± 0.3	6.9 ± 2.1	8	7.3 ± 0.7	8
DB1465	6.9 ± 1.9	71.4 ± 4.4	10	334 ± 11	48
DB1645	14.5 ± 2.8	233 ± 43	16	610 ± 60	42
DB1680	266 ± 21	591 ± 43	2	341 ± 11	1
DB1692	17.6 ± 4.4	436 ± 41	25	344 ± 14	20
Pentamidine	0.6 ± 0.3	0.9 ± 0.2	1.5	167 ± 10	278

a *n* = 3.

b RF, resistance factor (ratio of the IC_50_ measured against the *tbat1^−/−^* and B48 cell lines to its value obtained for the wild type).

**FIG 1:**
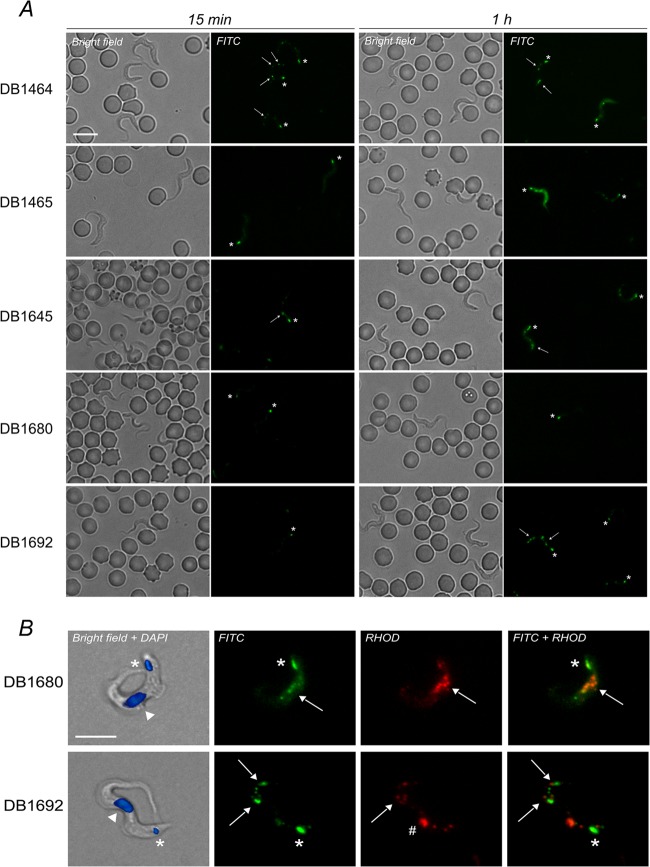
(A) Fluorescence images of infected rat blood incubated *ex vivo* with the five green fluorescent diamidines (note that the parasite position can change between the bright-field and corresponding fluorescence micrographs), 40× objective. (B) *In vitro* trypanosomes treated with DB1680 and DB1692 (50 μM, 1 h, 37°C), 100× objective; 4′,6-diamidino-2-phenylindole (DAPI) (20 μM) was used as a DNA counterstain. Asterisk, kinetoplast; arrow, cytoplasmic corpuscles, possibly acidocalcisomes; arrowhead, nucleus; hash mark, possibly lysosome. Bar, 10 μm.

All five diamidines stained live T. b. brucei in infected rat blood films using a standard fluorescein isothiocyanate (FITC) filter set (excitation, band-pass, 450 to 490 nm; beam splitter, 510 nm; and emission, band-pass, 515 to 565 nm) and a Zeiss Axioplan fluorescence microscope (Fig. 1A). Under our experimental conditions (*ex vivo* incubation for 15 min at 37°C, with 50 μM [each] fluorophore), all five diamidines provided specific staining of trypanosomes without labeling erythrocytes or white blood cells. Staining of the parasite nucleus was not observed, but all compounds selectively accumulated inside the kinetoplast (the parasite's mitochondrial genome, at the posterior of the cell) and other cytoplasmic organelles, spread throughout the cell body and also visualized at longer wavelengths (Fig. 1B). These were likely to be acidocalcisomes, previously observed to be stained by other diamidines (15). For DB1692, a prominent red fluorescent spot, putatively identified as the lysosome, also appeared in a region between the nucleus and the kinetoplast after 1 h of treatment. Under the FITC filter, parasites were readily detectable among blood cells based on their movement and fluorescent dots (especially the bright kinetoplast). Cell labeling was achieved only by incubating at room temperature or 37°C but not at 4°C. This temperature sensitivity could lead to variability in field tests using ambient temperature in different sites; hence, the use of temperature-controlled devices (e.g., water baths or heating blocks) might be required in such settings. Although the fluorescence intensity increased with the time of incubation for all probes, the overall yields remained below those identified with DB75 and could not be improved by increasing the fluorophore concentration. The low emission intensity of the new compounds could be due to their intrinsically low fluorescence yields (Table 1) but also to the discrepancy between their excitation maxima, all close to 400 nm, and the standard FITC filter wavelength utilized for microscopy. Hence, the use of these specific fluorophores with these particular filters may not be directly applicable to HAT diagnosis due to the risk of false negatives. However, these same compounds with customized filters or further derivatives with higher fluorescence yields could be considered as probes for the specific staining of trypanosomes using existing LED-based fluorescence microscopes.

Despite the compounds' partial dependence on the P2 transporter for uptake, they did not distinguish wild-type and *tbat1*^−/−^ knockout line by fluorescence microscopy (50 μM for incubations ranging from 15 min to up to 2 h), indicating that uptake routes independent of P2 play important roles in their internalization when used under these conditions.

Implementation of fluorescence tests for trypanosome detection in human fluids must rely on affordable, efficient and preferably specific markers. This work shows the feasibility of generating cheap fluorophores, emitting at differential wavelengths, by simple addition of cyclic groups within the chemical structure of diamidines, the precursor of which, DB75, is fluorescent only in the UV region. The exploitation of surface transporters peculiar to trypanosomes is a straightforward way to specifically target fluorophores to these parasites, and improved compounds may be developed following this approach to target both human and animal infective trypanosomes.

## Supplementary Material

Supplemental material
