# Supplementary material for: Green Fluorescent Diamidines as Diagnostic Probes for Trypanosomes
Source: Antimicrob Agents Chemother. 2014 Mar;58(3):1793–6. doi: 10.1128/AAC.02024-13 (PMC3957857; doi:10.1128/AAC.02024-13)
Supplement: Supplemental material [file supp_58_3_1793__index.html]

Green Fluorescent Diamidines as Diagnostic Probes for Trypanosomes — Supplemental material 

# Green Fluorescent Diamidines as Diagnostic Probes for Trypanosomes

## Supplemental material

**Files in this Data Supplement:**

- Supplemental file 1 -

  Description of the synthesis and chemical characterization of the five fluorescent diamidines.

  PDF, 151K
