# Supplementary material for: Green Fluorescent Diamidines as Diagnostic Probes for Trypanosomes
Source: Antimicrob Agents Chemother. 2014 Mar;58(3):1793–6. doi: 10.1128/AAC.02024-13 (PMC3957857; doi:10.1128/AAC.02024-13)
Supplement: Supplemental material [file AAC.02024-13_zac003142660so1.pdf]

SUPPLEMENTAL MATERIAL

Chemistry experimental

Melting points were recorded using a Thomas-Hoover (Uni-Melt) capillary melting point apparatus and are uncorrected. TLC analysis was carried out on silica gel 60 F_{254} precoated aluminum sheets and detected under UV light. ^{1}H and ^{13}C NMR spectra, were recorded employing a Varian Unity Plus 300 spectrometer or Bruker 400 MHz, using the indicated solvents, and chemical shifts (δ) are in ppm relative to TMS as internal standard. Mass spectra were recorded on a VG analytical 70-SE spectrometer. Elemental analyses were obtained from Atlantic Microlab Inc. (Norcross, GA). The compounds reported as salts frequently analyzed correctly for fractional moles of water and/or other solvents; in each case ^{1}H NMR spectra were consistent with the presence of water or organic solvent(s). All chemicals and solvents were purchased from Aldrich Chemical Co., VWR, Frontier Scientific or Combi-Blocks, Inc. The bis-nitriles required for synthesis of DB1464, DB1465 and DB1645 have been previously reported (1) and were used as described below. The synthesis of the bis-nitriles needed to prepare DB1680 and 1692 are outlined below. Brief synthetic Schemes are provided with each compound set.

5,5’’’’-Dicyano-2,2’:5’,2’’:5’’,2’’’:5’’’,2’’’’-quinguefuran.

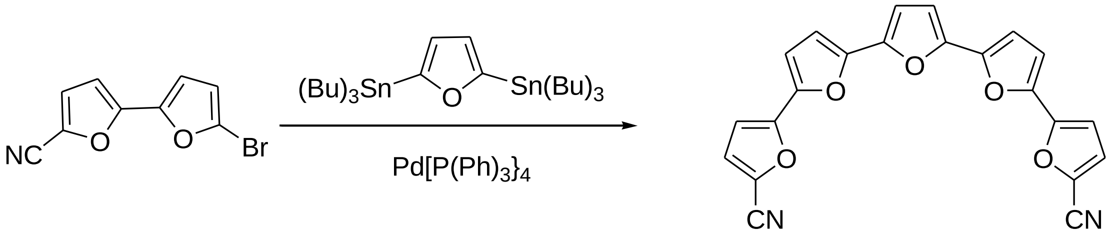

A mixture of 5'-bromo-2,2'-bifuran-5-carbonitrile (1) (1.19 g, 5 mmol), 2,5-bis(tri-*n*-butylstannyl)furan (1.6 g, 2.5 mmol) and tetrakis(triphenylphosphine)palladium(0) (125 mg) in dry 1,4-dioxane (40 ml) was heated under nitrogen at reflux (100-110 ^{o}C) for 24 h. The

precipitate which formed was collected, washed with hexanes and recrystallized from DMF to afford the product in 76% yield, mp 237-239 °C. ^{1}H NMR (DMSO-d_6); δ 7.08-7.23 (m, 8H), 7.78 (s, 2H). ^{13}C NMR (DMSO-d_6); δ 149.3, 145.8, 144.6, 143.3, 125.7, 124.0, 112.0, 109.7, 109.0, 107.7, 107.5. Anal. Calcd. for $C_{22}H_{10}N_2O_5$: C, 69.11; H, 2.64. Found: C, 68.83; H, 2.87.

5-Cyano-5”-(4-cyanophenyl)-2,2’:5’,2’’-terfuran.

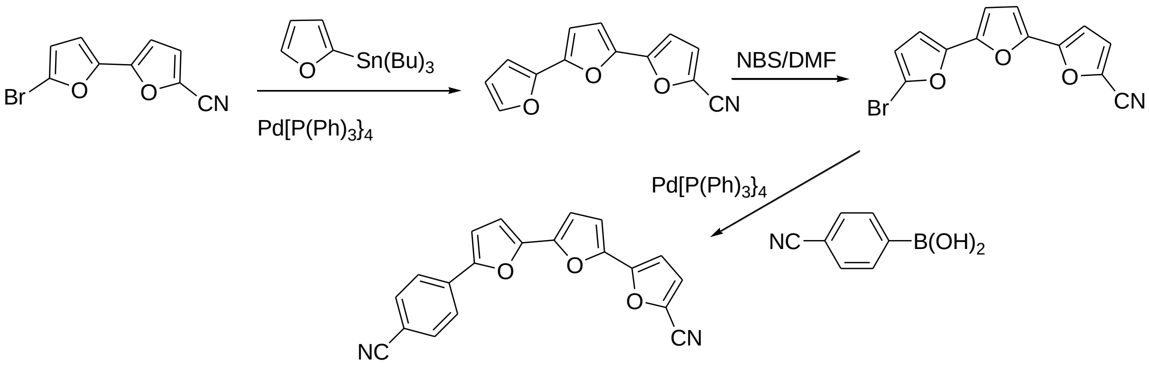

A similar procedure described for 5,5’’’’-dicyano-2,2’:5’,2’’:5’’,2’’’:5’’’,2’’’’-quinguefuran was used by employing 5'-bromo-2,2'-bifuran-5-carbonitrile (1) (1 equiv) and 2-(tri-*n*-butylstannyl)furan (1 equiv) to furnish 5-cyano-2,2’:5’,2’’-terfuran in 83% yield, mp 123.5-124 °C (EtOH). ^{1}H NMR (DMSO-d_6); δ 6.66-6.67 (m, 1H), 6.89-6.91 (m, 2H), 7.05 (d, J = 3.6 Hz, 1H), 7.17 (d, J = 3.6 Hz, 1H), 7.75 (d, J = 3.6 Hz, 1H), 7.81-7.82 (m, 1H). ^{13}C NMR (DMSO-d_6); δ 149.4, 146.6, 144.5, 143.8, 142.8, 125.6, 123.9, 112.1, 111.9, 111.7, 107.8, 107.4, 107.1. MS (EI) m/e (rel.int.); 225 (M^+, 30), 196 (6), 169 (15), 140 (55), 51 (100). The product was used directly in the next step.

To a solution of cyano-2,2’:5’,2’’-terfuran (2.25 g, 10 mmol) in DMF (25 ml) was added portionwise *N*-bromosuccinimide (1.78 g, 10 mmol) with stirring. The reaction mixture was stirred overnight at room temperature, then poured onto cold-water. The precipitate which formed was collected, washed with water and dried to give 5-bromo-5”-cyano-2,2’:5’,2’’-

terfuran in 92% yield, mp 154-155 ^{o}C. ^{1}H NMR (DMSO-d_6); δ 6.79 (d, J = 3.6 Hz, 1H), 6.93-6.96 (m, 2H), 7.07 (d, J = 3.6 Hz, 1H), 7.17 (d, J = 3.6 Hz, 1H), 7.75 (d, J = 3.6 Hz, 1H). ^{13}C NMR (DMSO-d_6); δ 149.2, 146.5, 145.3, 143.1, 125.6, 124.0, 122.5, 114.2, 111.9, 111.7, 109.9, 108.5, 107.5. MS (EI) m/e (rel.int.); 303, 305 (M^+, 8, 11), 224 (15), 196 (100). The product was used directly in the next step.

To a stirred solution of 5-bromo-5”-cyano-2,2’:5’,2’’-terfuran (1.51 g, 5 mmol), and tetrakis(triphenylphosphine) palladium (288 mg) in toluene (20 ml) under a nitrogen atmosphere was added 10 ml of a 1 M aqueous solution of $NaHCO_3$ followed by 4-cyanophenylboronic acid (875 mg, 6 mmol) in 5 ml of methanol. The vigorously stirred mixture was warmed to 80 ^{o}C for 24 h, then cooled, and the precipitate was filtered and recrystallized from DMF to afford the bis-nitrile in 67% yield, mp 241-243 $^{\circ}$C. ^{1}H NMR (DMSO-d_6); δ 7.08-7.14 (m, 3H), 7.24 (d, J = 4.4 Hz, 1H), 7.44 (d, J = 4.4 Hz, 1H), 7.78 (d, J = 4.4 Hz, 1H), 7.93 (d, J = 8.8 Hz, 2H), 7.98 (d, J = 8.8 Hz, 2H). ^{13}C NMR (DMSO-d_6); δ 151.5, 149.3, 146.0, 145.5, 143.5, 133.4, 133.04, 133.00, 128.1, 125.7, 124.1, 112.0, 111.7, 111.3, 110.0, 109.7, 109.3, 107.6. MS (EI) m/e (rel.int.); 326 (M^+, 25), 270 (5), 214 (12), 140 (75), 75 (100). Anal. Calcd. for $C_{20}H_{10}N_2O_3$: C, 73.62; H, 3.09. Found: C, 73.25; H, 3.18.

Bis-amidino oligochalcophenes.

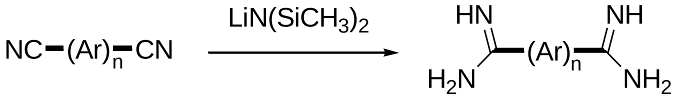

5,5’-Bis(5-amidino-2-furanyl)-2,2’-bifuran hydrochloride (DB1464).

The bis-nitrile 5,5’-bis(5-cyano-2-furanyl)-2,2’-bifuran (1) (316 mg, 1 mmol), suspended in freshly distilled THF (5 ml), was treated with lithium trimethylsilylamide (1M solution in THF, 6 ml, 6 mmol) and the reaction was allowed to stir for 48 hr. The reaction mixture was

then cooled to 0 °C to which was added HCl saturated ethanol (60 ml) whereupon a precipitate started forming. The mixture was left to run overnight whereafter it was diluted with ether and the resultant solid was collected by filtration. The diamidine was purified by neutralization with 1N NaOH followed by filtration of the resultant solid and washing with water (3×). Finally, the free base was stirred with ethanolic HCl overnight, diluted with ether, and the solid formed was filtered and dried to give the diamidine salt DB1464 in 62% yield, mp >300 °C. ^{1}H NMR (D_2O/DMSO-d_6): δ 7.02 (d, J = 3.6 Hz, 2H), 7.05 (d, J = 3.9 Hz, 2H), 7.19 (d, J = 3.6 Hz, 2H), 7.74 (d, J = 3.9 Hz, 2H). ^{13}C NMR (D_2O/DMSO-d_6); δ 153.8, 149.9, 146.3, 144.3, 140.1, 121.7, 112.9, 110.2, 109.4. MS (ESI) m/e (rel. int.): 351 (M^+ +1, 78), 176 (100). HRMS calcd. for $C_{18}H_{15}N_4O_4$: 351.1093. Observed: 351.1091. Anal. Calcd. For $C_{18}H_{14}N_4O_4$-2.0HCl-2.4H_2O: C, 46.34; H, 4.49; N, 12.01. Found: C, 46.09; H, 4.26; N, 11.68.

5,5’-Bis(5-amidino-2-furanyl)-2,2’-bithiophene hydrochloride (DB1465).

The diamidine DB1465 was prepared from 5,5’-bis(5-cyano-2-furanyl)-2,2’-bithiophene following the procedure used for DB1464 in a 60% yield, mp >300 °C. ^{1}H NMR (D_2O/DMSO-d_6): δ 7.11 (d, J = 3.9 Hz, 2H), 7.49 (d, J = 3.9 Hz, 2H), 7.72 (d, J = 3.9 Hz, 2H), 7.79 (d, J = 3.9 Hz, 2H). ^{13}C NMR (D_2O/DMSO-d_6); δ 153.7, 153.5, 139.8, 138.0, 130.3, 128.7, 126.6, 122.1, 109.6. MS (ESI) m/e (rel. int.): 383 (M^+ +1, 80), 192 (100). HRMS calcd for $C_{18}H_{15}N_4O_2S_2$: 383.0636. Observed: 383.0643. Anal. Calcd. For $C_{18}H_{14}N_4O_2S_2$-2.0HCl-1.7H_2O-0.25EtOH: C, 44.66; H, 4.23; N, 11.26. Found: C, 44.75; H, 3.99; N, 10.95.

5, 5’-Bis(5-amidino-2-thienyl)-2, 2’-bifuran hydrochloride (DB1645).

The diamidine DB1645 was prepared from 5, 5’-bis(5-cyano-2-thienyl)-2, 2’-bifuran following the procedure used for DB1464 in a 67% yield of dark reddish solid; mp >300 °C dec.; ^{1}HNMR (DMSO-d_6): 7.05 (d, J = 3.5 Hz), 7.27 (d, J = 3.5 Hz), 7.68(d, J = 4.0 Hz), 8.03 (d, J = 4.0 Hz), 9.15 (vbr, 4H), 9.38 (vbr, 4H); ^{13}CNMR (DMSO-d_6): 147.1, 145.2, 140.2,

138.9, 123.9, 114.4, 111.7, 110.0, 106.2; MS: m/e 383 (M^{+}+1); Anal. Calcd. For $C_{18}H_{14}N_4O_2S_2$-2HCl-1.5H_2O (482.4): C,44.81; H,3.96; N,11.61; Found: C,44.75; H,3.98; N,11.31.

5,5’’’’-Diamidino-2,2’:5’,2’’:5’’,2’’’:5’’’,2’’’’-quinguefuran hydrochloride (DB1680).

The diamidine was prepared from 5,5’’’’-Dicyano-2,2’:5’,2’’:5’’,2’’’:5’’’,2’’’’-quinguefuran following the procedure used for DB1464 in a 65 % yield. mp 297-300 ^{o}C. ^{1}H NMR (D_2O/DMSO-d_6); δ 7.03-7.05 (m, 4H), 7.12 (d, J = 4.0 Hz, 2H), 7.31 (d, J = 4.0 Hz, 2H), 7.94 (d, J = 4.0 Hz, 2H). ^{13}C NMR (D_2O/DMSO-d_6); δ 153.6, 150.0, 146.6, 145.3, 144.1, 140.2, 121.7, 113.0, 110.4, 109.7, 109.3. MS (ESI) m/e (rel.int.); 417 (M^{+} + 1, 65), 400 (4), 351 (10), 275 (4), 242 (40), 209 (100). HRMS calcd. for $C_{22}H_{17}N_4O_5$: 417.1199. Observed: 417.1184. Anal. Calcd. for $C_{22}H_{16}N_4O_5$-2.0HCl-1.75H_2O: C, 50.73; H, 4.16; N, 10.75. Found: C, 50.65; H, 4.02; N, 10.56.

5-Amidino-5”-(4-amidinophenyl)-2,2’:5’,2’’-terfuran hydrochloride (DB1692).

The diamidine was prepared from 5-cyano-5”-(4-cyanophenyl)-2,2’:5’,2’’-terfuran following the procedure used for DB1464 in a 73 % yield mp >300 ^{o}C. ^{1}H NMR (D_2O/DMSO-d_6); δ 7.09 (d, J = 3.6 Hz, 1H), 7.14 (d, J = 3.6 Hz, 1H), 7.19 (d, J = 3.6 Hz, 1H), 7.33 (d, J = 3.6 Hz, 1H), 7.47 (d, J = 3.6 Hz, 1H), 7.96-8.05 (m, 5H). ^{13}C NMR (D_2O/DMSO-d_6); δ 164.8, 153.2, 151.7, 149.0, 146.1, 145.5, 143.8, 140.1, 134.1, 129.0, 126.4, 123.6, 121.0, 112.3, 111.4, 109.9, 109.3, 108.6. MS (ESI) m/e (rel.int.); 361 (M^{+} + 1, 100), 275 (12), 242 (94). HRMS calcd. for $C_{20}H_{17}N_4O_3$: 361.1301. Observed: 361.1298. Anal. Calcd. for $C_{20}H_{16}N_4O_3$-2.0HCl-2.25H_2O-0.25C_2H_5OH: C, 50.73; H, 4.98; N, 11.54. Found: C, 50.76; H, 4.54; N, 11.10.

REFERENCE

1. **Ismail MA.** 2006. An efficient synthesis of 5 '-(4-cyanophenyl)-2,2 '-bifuran-5-carbonitrile and analogues. J. Chem. Res. S**:**733-737.
